# Further Evidence for Reliability and Validity of the Triarchic Psychopathy Measure in a Forensic Sample and a Community Sample

**DOI:** 10.1007/s10862-016-9567-5

**Published:** 2016-08-15

**Authors:** Josanne D. M. van Dongen, Laura E. Drislane, Henk Nijman, Sabrina E. Soe-Agnie, Hjalmar J. C. van Marle

**Affiliations:** 1 Institute of Psychology, Erasmus University Rotterdam, P.O. Box 1738, 3000 DR Rotterdam, The Netherlands; 2 Department of Psychology, Florida State University, Tallahassee, Florida USA; 3 Behavioural Science Institute, Radboud University Nijmegen, Nijmegen, The Netherlands; 4 Tactus Addiction Center, Apeldoorn, The Netherlands; 5 Department of Psychiatry, Erasmus Medical Center, Rotterdam, The Netherlands

**Keywords:** Pychopathy, Triarchic Psychopathy Measure, ROC curve, Discriminant validity, Forensic patients

## Abstract

Psychopathy is often described as a constellation of personality characteristics encompassing features such as impulsivity and antisociality, and a lack of empathy and guilt. Although the use of self-reports to assess psychopathy is still debated, there are distinct advantages to such measures and recent research suggests that they may not be as problematic as previously thought. This study further examined the reliability and validity of the Triarchic Psychopathy Measure (TriPM) in a community sample (*N* = 496) and forensic psychiatric patient sample (*N* = 217). Results indicated excellent internal consistencies. Additionally, the TriPM total and subscale scores related as expected to different subscales of the Psychopathic Personality Inventory –Revised (PPI-R) and to the Reactive and Proactive Aggression Questionnaire, reflecting good construct validity. Most importantly, ROC curve analyses showed that the TriPM evidenced better discrimination between the community sample and forensic psychiatric patients than the PPI-R. The current study extends the existent evidence demonstrating that the TriPM can be used as an efficient self-report instrument.

## Introduction

Psychopathy is considered to entail a constellation of personality characteristics including impulsive and antisocial behavior, callous use of others, lack of guilt, and shallow affect (Cleckley 1976). A number of different models and measures have been developed to describe and assess this construct. One conceptualization that has become increasingly influential in the psychopathy literature in recent years (see Patrick and Drislane 2014 for a review) is the triarchic model of psychopathy (Patrick et al. 2009). The current study evaluated the convergent validity and clinical utility of the Dutch translation of the Triarchic Psychopathy Measure (TriPM; Patrick 2010) – a self-report operationalization of the triarchic model constructs of boldness, meanness, and disinhibition – in two samples consisting of members of the general community and forensic inpatients.

## Conceptions of Psychopathy

Cleckley’s classic work *The Mask of Sanity* (Cleckley 1976) served as the foundation for modern conceptions and measures of psychopathy. For example, Hare (1991, 2003) used Cleckley’s list of 16 clinical criteria as a basis to formulate a diagnostic instrument for the assessment of psychopathic personality. The Revised version of Hare’s Psychopathy Checklist (PCL-R), an interview and file-based assessment instrument, is still regarded as the most well-validated measure for assessing psychopathy in forensic and correctional settings (but see Boduszek and Debowska 2016). The PCL-R measures psychopathy in terms of two broad factors: Factor 1, encompassing Affective and Interpersonal facets of psychopathy, and Factor 2, encompassing Antisocial and Lifestyle facets. A rich empirical literature has been amassed on the PCL-R and its variants (see Neumann et al. 2014 for a review); however, like any assessment instrument, it has certain limitations. One is that several of its items refer directly to criminal activity, which makes the PCL-R most appropriate for use with correctional or forensic samples. Another is that the PCL-R is interview-based, and requires access to collateral (i.e., archival file) information. Therefore, its very time consuming to administer, and impractical for large-scale data collection efforts.

In light of these limitations, alternative self-report based instruments have been developed for assessing psychopathy. However, the use of self-report measures to assess psychopathy has been debated due to concerns that psychopathy may be associated with ‘faking good,’ a tendency to present oneself in a socially desirable manner–which could operate to invalidate assessments of psychopathy using self-report measures. However, recent studies have demonstrated that self-report measures correlate in meaningful ways with interview-based measures of psychopathy such as the PCL-R (Patrick 2010), and are negatively rather than positively related to social desirability (Verschuere et al. 2014), suggesting that high-psychopathy individuals are actually less likely to produce overly positive self-reports.

The most widely used instrument for assessing psychopathy in the domain of self-report is the Psychopathic Personality Inventory (PPI; Lilienfeld and Andrews 1996). The PPI was developed to index the core personality traits associated with conceptions of psychopathy advanced by Cleckley 1976 and others without items explicitly referencing criminal behavior. The PPI has been extensively validated in undergraduate, community, and correctional settings (see Liliengeld and Fowler 2006, for a review). Factor analytic studies have identified two higher-order factors accounting for covariance among the PPI’s eight primary scales (Benning et al. 2005). The first factor, Fearless-Dominance, encompasses lower-order subscales of Social Potency, Fearlessness, and Stress Immunity reflecting, respectively, tendencies toward social dominance and persuasiveness, an ability to remain calm in stressful situations, and a lack of situational fear. The PPI Fearless-Dominance construct is likely to be particularly relevant to conceptualization and measurement of psychopathy in noncriminal samples, including identification of individuals with psychopathic tendencies who ascend to positions of leadership and influence in society (Babiak and Hare 2006; Lilienfeld et al. 2012b). The second factor, Impulsive-Antisociality (referred to as Self-centered Impulsivity in the revised version of the PPI (PPI–R; Lilienfeld and Widows 2005), encompasses subscales of Machiavellian Egocentricity, Rebellious Nonconformity, Blame Externalization, and Carefree Nonplanfulness, reflecting tendencies to be self-centered, oppositional and reckless, blameful of others, and impulsive. The remaining PPI subscale, Coldheartedness, reflects tendencies toward callousness and a lack of guilt. It does not load substantially onto either factor and was designated as a third factor on the PPI–R (Lilienfeld and Widows 2005).

One newer model of psychopathy is the Triarchic conceptualization of psychopathy (Patrick et al. 2009). The Triarchic model was advanced as a framework for integrating the major themes discussed in historic and contemporary accounts of psychopathy, and a means for organizing empirical findings related to the understanding and assessment of psychopathy. The triarchic model posits that psychopathy reflects three distinctive etiological phenotypic constructs, namely ‘boldness’, ‘meanness’, and ‘disinhibition’. *Boldness* is defined as a phenotypic manifestation of an underlying genotypic predisposition toward fearlessness (Patrick 2010). Individuals high on boldness report few specific fears, have the ability to remain calm under stressful circumstances, and have a high level of social influence and dominance. *Meanness* is viewed as a more malignant phenotypic expression of fearlessness, combined with a lack of affiliative capacity, and manifests as lack of empathy (e.g. callousness), low social closeness, and cruel behavior towards humans and animals. The third construct, *disinhibition,* is defined as the common dispositional tendency underlying externalizing conditions of differing types and entails weak inhibitory control and deficient affect regulation. Individuals high in disinhibition lack planning, are impulsive, and have difficulty controlling emotions and constraining behaviors.

An inventory that was developed specifically to operationalize these constructs in domain of self-report is the Triarchic Psychopathy Measure (TriPM; Patrick 2010). The TriPM is a measure that is developed to assess the different constructs within the triarchic psychopathy measure, but not designed to capture a structural model. Sellbom and Phillips (2013) reported on the psychometric properties and provided initial evidence for the construct validity of the TriPM in two samples consisting of undergraduate students and criminal offenders. The TriPM accounted for variance in other psychopathy measures in a manner consistent with theoretical expectations, providing support for convergent validity of the TriPM. Theory-consistent relations with other relevant criterion measures were also evident. For example, the TriPM Boldness was preferentially associated with narcissism, thrill/adventure seeking, and low behavioral inhibition system functioning, whereas TriPM Meanness was more associated with Machiavellianism, low empathy, and low behavioral inhibition system, and Disinhibition with impulsivity and fun-seeking. Other studies have provided further evidence regarding the internal psychometric properties and criterion-related validity of the TriPM (e.g., Drislane et al. 2014a; Stanley et al. 2013).

The aim of the current study was to extend what is known about the reliability and validity of TriPM, using a Dutch-language translation of the instrument and two distinctive participant samples: adults from the general community and forensic psychiatric patients. Along with evaluating the internal consistencies of the TriPM and its subscales in these samples, we also evaluated its validity in relation to novel criterion measures including the Reactive and Proactive Aggression Questionnaire (RPQ; Cima et al. 2013). Based on prior research as described above, we predicted that the TriPM would show good psychometric properties and meaningful relations with external criteria. A further aim was to use Receiver Operating Characteristic (ROC) analyses to evaluate the ability of the TriPM and PPI-R to discriminate between participants in the two study samples (community adults, forensic patients).

## Method

### Participants

The data were obtained from two separate samples comprising a total of 755 participants, a forensic psychiatric sample (*N* = 217; 90% males) and a sample drawn from the general population (*N* = 496; 49% males). The forensic psychiatric sample is comprised of patients from two different facilities; a high security forensic psychiatric center and a forensic addiction clinic. The majority of patients from the high security psychiatric sample had a primary diagnosis of schizophrenia or other psychotic disorder (35.3%) or antisocial personality disorder (20.7%). Although 3.3% of data concerning diagnosis was missing in the patients from the forensic addiction clinic, all the rest had a diagnosis of substance abuse or dependency. Mean age of the forensic sample was 38.31 (*SD* = 9.16) and for the community sample mean age was 27.70 (*SD* = 13.09). For 10 participants in the community sample, information concerning age was not available. Participants were at least 18 years of age. Participants were required to be able to speak the Dutch language. Additional exclusion criterion for the community sample was presence of mental disorder.

For ethnic composition of the samples, see Table 1. For means for the different measures in the community and forensic sample, see Table 2.

**Table 1:** Ethnic composition of the community and forensic psychiatric samples

	Community sample (*n* = 385)	Forensic Psychiatric sample (*n* = 296)
Ethnicity		
West-European	92.8%	66.2%
East-European	0 .9%	0 .7%
South-European	1.5%	5.7%
Middle East	0 .9%	0. 0%
North-African	0 .4%	4.3%
South-African	0 .4%	9.7%
Middle and South American	2.0%	13.4%
Asian	1.1%	0. 0%

**Table 2:** Means and standard deviations for study measures in community and forensic psychiatric samples

		Community sample	Forensic Psychiatric sample
TriPM ^a^	Total	54.90 (16.89)	73.36 (21.68)
	Boldness	31.08 (8.14)	30.59 (9.22)
	Meanness	12.26 (7.79)	16.12 (9.58)
	Disinhibition	11.46 (7.80)	26.66 (12.02)
PPI-R ^b^	Total	285.71 (34.50)	302.32 (40.84)
	PPI-I	113.35 (18.30)	113.70 (21.88)
	PPI-II	127.73 (23.38)	154.55 (25.03)
	PPI-III	34.63 (6.29)	34.11 (7.94)
RPQ ^c^	Total	9.94 (5.30)	40.61 (8.73)
	RPQre	8.29 (3.73)	22.11 (4.41)
	RPQpro	1.63 (1.96)	18.50 (5.26)

*TriPM* Triarchic Psychopathy Measure, *PPI-R* Psychopathic Personality Inventory-Revised, *PPI-I* Fearless Dominance, *PPI-II* Self-Centered Impulsivity, *PPI-III* Coldheartedness, *RPQ* Reactive-Proactive Aggression Questionnaire, *RPQre* Reactive Aggression, *RPQpro* Proactive Aggression

^a^community *n* = 496, forensic *n* = 296

^b^community *n* = 360, forensic psychiatric *n* =132

^c^community *n* = 93, forensic *n* = 164

### Measures

**Triarchic Psychopathy Measure** (TriPM; Patrick 2010). We used the Dutch translation of the TriPM (Soe-Agnie et al. 2011). The TriPM consists of 58 items, each rated on a 4-point Likert-scale (3 = *true*, 2 = *somewhat true*, 1 = *somewhat false*, 0 = *false*), with scoring reversed for items worded in the direction of lower psychopathy. The items of the TriPM form three distinct scales, which index the three phenotypic constructs delineated by the triarchic model: Boldness, Meanness, and Disinhibition. The Boldness scale contains 19 items that index tolerance for uncertainty or danger, interpersonal dominance, and a low degree of fear. The TriPM Meanness scale also includes 19 items, which index traits such as callousness, aggression, and a lack of empathy. The Disinhibition scale, consisting of 20 items, reflects tendencies toward impulsivity, a lack of goal-oriented behavior and planning, and alienation.

The items of the TriPM were translated into Dutch from the original U.S. version and back-translated into English. If there was discrepancy in item phrasing after back-translation, the

Dutch item was rephrased in order to assess the essence of the given trait, all according to standard procedures.

**Psychopathic Personality Inventory – Revised** (PPI-R; Lilienfeld and Widows 2005). The Dutch translation of the Psychopathic Personality Inventory-Revised (PPI-R; Jelicic et al. 2004) consists of 154 items rated on a 4-point Likert-scale (1 = *false*, 2 = *mostly false*, 3 = *mostly true*, and 4 = *true*). As noted earlier, the items of the PPI-R are organized into eight subscales that demarcate three factors, the Fearless-Dominance (PPI-R-I), Impulsive –Antisociality (PPI-R-II) and Coldheartedness (PPI-R-III). However, the usefulness of this last factor has been debated in some studies (see Marcus et al. 2012). Uzieblo et al. (2010) found, in a community sample, that the Dutch PPI-R shows good convergent, discriminant, and external validity, but the factor structure in this sample differed from the structure reported in American adult community samples (e.g., Benning et al. 2003, Benning et al. 2005; Lilienfeld and Widows 2005; Patrick et al. 2006).

**Reactive-Proactive Aggression Questionnaire** (RPQ; Raine et al. 2006). The Dutch translation of the RPQ (Cima et al. 2013) was used to measure reactive and proactive aggression. The RPQ consists of 23 items; 12 items make up the proactive subscale (i.e. instrumental aggression) and 11 items make up the reactive subscale (i.e. impulsive aggression). The items are scored on a 3-point Likert scale ranging from 0 (*never*) to 2 (*most of the time*). Cima et al. (2013) found that the Dutch version of the RPQ, has the same two-factor structure as the original version and has good test-retest reliability, as well as good criterion, construct, and convergent validity.

### Procedure

Participants comprising the general population sample were recruited via social (online) networks, or participated for course credit. Forensic patients were recruited from two different forensic mental institutions in the Netherlands and received a snack item for participation. Because participants were part of different studies and later merged into this current dataset, the specific questionnaires administered in each study differed to some extent across participants. However, all participants in the present study completed the TriPM. From the community sample, 93 participants filled out the RPQ and 360 filled out the PPI-R. From the forensic psychiatric sample, 164 participants filled out the RPQ and 132 filled out the PPI-R. Before completing the packet of questionnaires, all participants received information regarding the purposes of the study and provided written informed consent. Subsequently, all participants filled out some demographic questions (i.e. gender, age, cultural background, education, marital status, and income). After completing demographic information, the participants filled out the other questionnaires (TriPM, RPQ and PPI-R).

### Statistical Analysis

Data were analyzed using SPSS version 21.0. For examining the psychometric properties of the scale, internal consistencies of the TriPM as whole and its subscales were evaluated using Cronbach’s alpha reliability coefficients.

To evaluate the construct validity of the TriPM, correlations were examined between scores for the subscales of the TriPM and criterion measures consisting of RPQ scale scores and the PPI-R factor scores.

Finally, to test for discriminant validity, area under the curve (AUC) of the ROC curve were calculated for the TriPM, PPI-R and the different subscales in discriminating between the community sample and the forensic sample. In ROC analysis, sensitivity and false-positive rate are plotted, resulting in an ROC curve. If the sensitivity of a scale equals its false-positive rate at each possible cut-off point, the ROC curve will appear as a diagonal line. This *line of no information* specifies that the particular scale is unable to discriminate between the general population sample and the forensic sample. Conversely, the better a scale differentiates between the two samples, the more the ROC curve will deviate in a positive direction from the line of no information. The AUC represents the probability that a randomly selected participant from the target group (e.g., forensic patients) will have a higher score on the scale than a randomly selected participant from the comparison group (e.g., participant from the general population) (Hanley and McNeil 1982). AUCs can range from.50 (the line of no information) to 1.00 (a perfect diagnostic sign). An advantage of ROC analysis is that the AUCs of various scales (or subscales) can be compared directly to determine their relative diagnostic efficiencies.

## Results

### TriPM Scale Intercorrelations and Reliabilities

In both the forensic and community sample, the intercorrelations between the Boldness and Meanness subscales were significant, but low/moderate (*r* = .308 and *r* = .195 respectively); the intercorrelations for the Boldness and Disinhibition subscales were non-significant in both samples, and the intercorrelations between the Meanness and Disinhibition subscales were significant and high for the forensic sample (*r* = .483) and the community sample (*r* = .594).

To test the reliability of the TriPM, internal consistencies were determined using Cronbach’s α. Internal consistency coefficients for TriPM scores were uniformly high. For the total scale, α’s for the community and forensic samples were .87 and .88, respectively. For the Boldness subscale, α’s were .83 and .86, respectively; for Meanness, α’s were .85 and .86; and for Disinhibition, α’s were .82 and .80.

### Construct Validity

To evaluate the criterion-related validity of the TriPM, correlations between scores on the TriPM, PPI-R, and RPQ were calculated (see Tables 3 and 4). Overall, the TriPM showed strong convergence with the PPI-R. As expected, total scores for both measures correlated highly with one another in both the community (*r* = .82) and forensic psychiatric samples (*r* = .76). Also, scores on subscales of the TriPM related to the Factors of the PPI-R as expected. That is, the TriPM Boldness scale related most strongly to PPI-R-I (fearless-dominance), with a lesser positive correlation evident for TriPM Meanness. TriPM Disinhibition related strongly to scores on PPI-R-II (impulsive-antisociality) in both samples, with TriPM Meanness correlating to a somewhat lesser degree (presumably as a function of inclusion of the PPI-R’s Machiavellian Egocentricity subscale; Drislane et al. 2014a). Further, as hypothesized, TriPM Meanness showed a strong association with scores on PPI-R-III (coldheartedness), with a smaller positive correlation evident for TriPM Meanness.

**Table 3:** Pearson’s correlations between the TriPM total and subscale scores and the PPI-R

Sample		PPI-R	PPI-R	PPI-R	PPI-R
		Total	I	II	III
Community (*n* = 360)	TriPM total	.82**	.57**	.67**	.37**
	Boldness	.54**	.79**	.12*	.26**
	Meanness	.66*	.30**	.62**	.46**
	Disinhibition	.52**	.06	.70**	.05
Forensic Psych. (*n* = 132)	TriPM total	.76**	.57**	.61**	.42**
	Boldness	.62**	.85**	.19*	.25**
	Meanness	.62**	.39**	.49**	.51**
	Disinhibition	.43**	.08	.60**	.12

*α<.05

**α<.001

**Table 4:** Pearson’s correlations between the TriPM total and subscale scores and the RPQ

Sample		RPQ Total	RPQ Reactive	RPQ Proactive
Community (*n*= 93)	TriPM total	.52**	.42**	.61**
	Boldness	.16	.20	.32
	Meanness	.52**	.62**	.41**
	Disinhibition	.48**	.53**	.41**
Forensic Psych. (*n* = 164)	TriPM total	.65**	.50**	.65**
	Boldness	.06	-.04	.13
	Meanness	.59**	.44**	.61**
	Disinhibition	.64**	.58**	.58**

*α<.05

**α<.001

TriPM scores were also correlated with scores on the RPQ aggression inventory. In each participant sample, total scores on the TriPM were related to RPQ total scores, as well as to both reactive and proactive aggression, with higher relations to proactive than reactive aggression. Boldness did not relate to either RPQ aggression scale in both samples. Additionally, Meanness and Disinhibition were significantly related to both forms of aggression, with Meanness related more strongly to proactive than reactive aggression in the forensic sample.

Because scores on Meanness and Disinhibition are moderately correlated, we further evaluated relations between these TriPM scales and aggression of the two types using regression analyses in the two samples. When Meanness and Disinhibition were entered together as predictors of each RPQ subscale in the community sample, results showed that Meanness correlated more strongly with proactive aggression (*β* = .469, *p* < .001) than Disinhibition did (*β* = .247, *p* = .039). Although both Disinhibition and Meanness were found to be predictors for reactive aggression, results were found to be non-significant. When Meanness and Disinhibition were entered together as predictors of each RPQ subscale in the forensic psychiatric sample, results showed that Meanness correlated more strongly with proactive aggression (*β* = .429, *p* < .001) than Disinhibition did (*β* = .358, *p* < .001), but Disinhibition was found to be more strongly related to reactive aggression (*β* = .476, *p* < .001) than Meanness (*β* = .203, *p* = .006). These complementary results may indicate that in the forensic psychiatric sample, Meanness and Disinhibition are related to different forms of aggression; Meanness is mainly associated with proactive aggression and Disinhibition is mainly associated to reactive aggression.

### Discriminative Ability of the TriPM

**Fig. 1:**
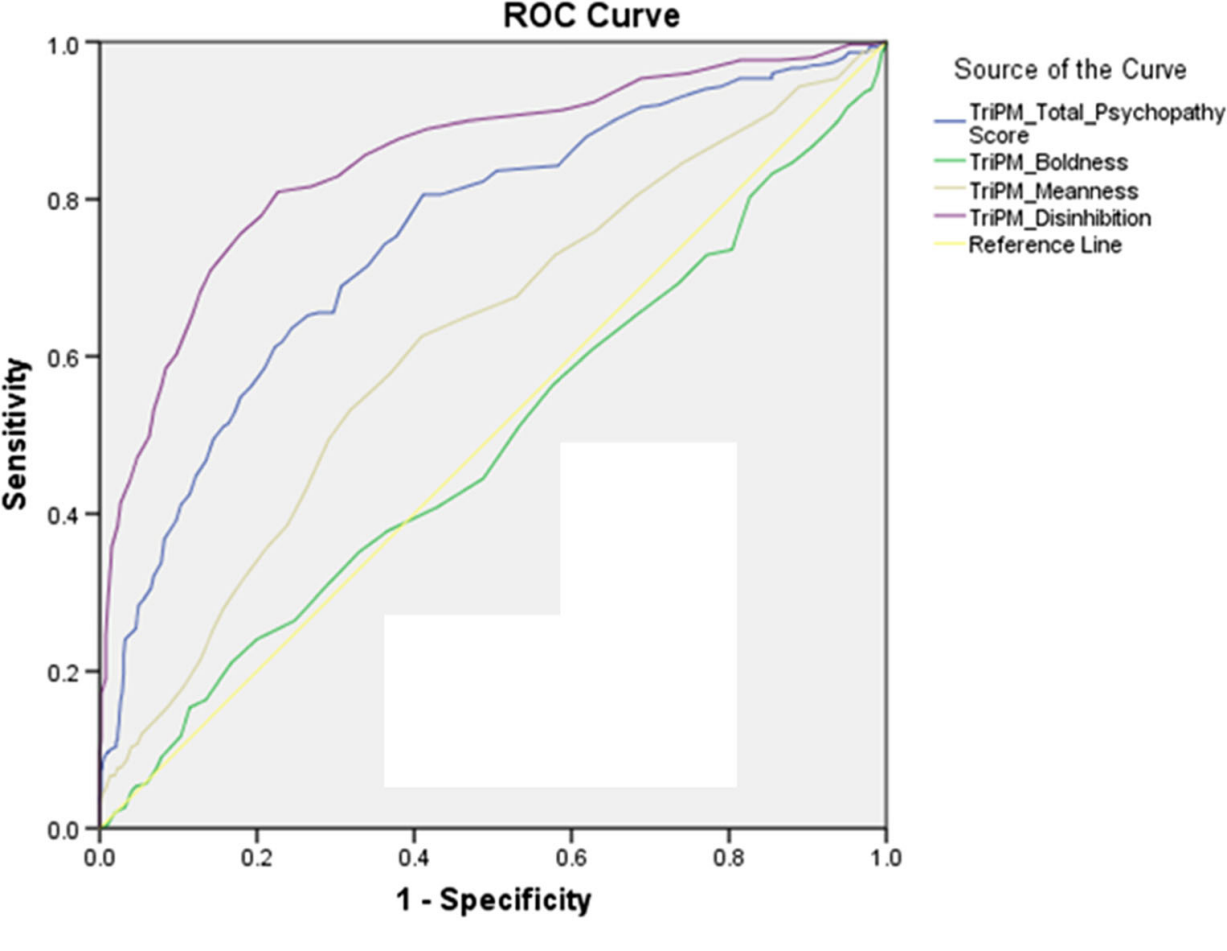
ROC curve for TriPM scores. AUC values for the total TriPM scores and the different TriPM subscales, reflecting their sensitivity and specificity for discriminating between the forensic psychiatric sample and the community sample

**Fig. 2:**
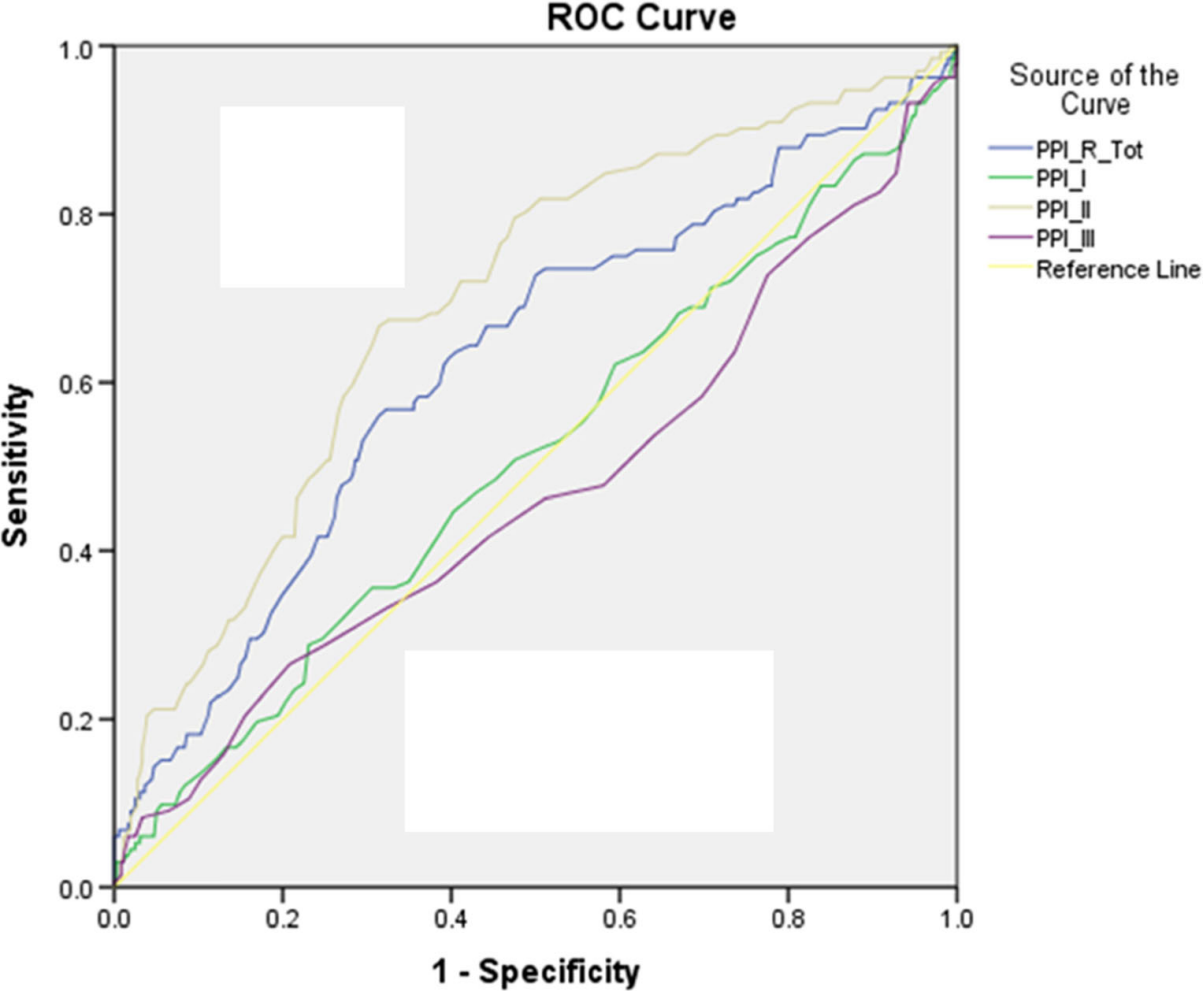
ROC curve for PPI-R scores. AUC values for the total PPI-R scores and the different PPI-R subscales, reflecting their sensitivity and specificity for discriminating between the forensic psychiatric sample and the community sample

The AUCs (95% confidence interval) of the ROC curves for the TriPM and PPI-R total scores and subscale scores were calculated (see Figs. 1 and 2). Overall, AUCs for the TriPM were higher than for the PPI-R. AUC for TriPM total scores was .753 (*p* = < .001; 95% CI = .718 –.788) and AUC for the PPI-R total scores was .635 (*p* = < .001; 95% CI = .567 –.682), indicating strong effectiveness of the total scores in distinguishing participants in the forensic psychiatric sample from those in the community sample.

For both measures, the Disinhibition scale of the TriPM and the Self-centered-Impulsivity subscale of the PPI-R (PPI-R-II) had the highest discriminant abilities. The Disinhibition subscale showed excellent discrimination (AUC = .851; *p* < .001; 95% CI = .822 – 879), while the Self-centered-Impulsivity subscale showed lower discrimination (AUC = .696; *p* < .001; 95% CI = .643 – .748).

While he AUC of the Meanness subscale was .629 and significant (*p* < .001; 95% CI = .579 - .659), AUC for the Coldheartedness scale (PPI-R-III) was non-significant (AUC = .471; *p* = .321; 95% CI = .410 – .532).

Both the Boldness subscale of the TriPM and the Fearless-Dominance subscale of the PPI-R (PPI-I) were not able to distinguish between the two participant samples. The Boldness subscale had an AUC of .489 (*p* = .608; 95% CI = .447 - .531), and the AUC of the PPI-R-I was .511 (*p* = .704; 95% CI = .452 - .571). These results indicate that Boldness and Fearless-Domainance tap aspects of psychopathy less related to clinical-pathologic status (Lilienfeld et al. 2012a; Miller and Lynam 2016; see also Widiger et al. 1996).

## Discussion

The present study further examined the reliability and validity of the TriPM in a forensic psychiatric sample and community sample. The results showed that across both samples, the instrument as a whole as well as its subscales (i.e. Boldness, Meanness and Disinhibition) had excellent internal consistencies, providing strong evidence that the TriPM is a reliable instrument. In addition, results also showed that in both samples, the TriPM showed good construct validity in terms of its relation with relevant criterion measures (i.e. the PPI-R and RPQ). TriPM total and subscale scores correlated positively with the PPI-R total scores and subscale scores in a manner consistent with observed relations between the PPI/PPI-R and the original English-language version of the TriPM (Drislane et al. 2014a; Sellbom & Phillips, 2013; Stanley et al. 2013). Additionally, as hypothesized, total scores on the TriPM as well as Meanness and Disinhibition subscale scores correlated with both reactive and proactive aggression, with Meanness to be more strongly related to proactive aggression and Disinhibition found to be more strongly related to reactive aggression. This finding is in line with the idea that callous-unemotional characteristics of psychopathy in particular are related to instrumental aggression (e.g. Cornell et al. 1996). In addition, in both samples scores on Boldness were unrelated to total or subscale scores on the RPQ, consistent with meta-analytic findings that fearless-dominant tendencies are largely unrelated to aggression (Miller and Lynam 2012). These results are partly in line with recent findings of Donnellan and Burt (2015) who found Boldness to be unrelated to aggression and both Meanness and Disinhibition to be related to proactive aggression, while reactive aggression was not related to either subscales of the TriPM.

Because of the ongoing debate on whether self-report measures can be validly used for assessing psychopathy, the second aim of the study was to determine the discriminative ability of the TriPM and the PPI-R by examining its ability to differentiate between forensic psychiatric and non-forensic samples. ROC curve analyses were used to examine the AUC values (i.e. sensitivity and specificity) of the TriPM and PPI-R total and subscale scores. Results showed that overall, the TriPM had better discriminant abilities than the PPI-R. There were some differences in terms of usefulness of the different subscales of the TriPM and PPI-R in differentiating forensic psychiatric and non-forensic groups. The Disinhibition and PPI-R-II subscales showed the best discrimination between the community sample and forensic psychiatric sample, followed by the Meanness scale of the TriPM. The Boldness, PPI-R-I and PPI-R-III by contrast, were found not to be useful for discriminating the two samples. Given that rates of externalizing psychopathology and antisocial behavior were markedly higher in the adjudicated forensic psychiatric sample compared to the general community sample, it makes sense that scales indexing disinhibitory and callous-aggressive tendencies would discriminate strongly between the two samples. Nonetheless, it is impressive that brief scale measures proved so discriminating, considering concerns that have been raised about self-report assessment with offenders.

Notably, the results of the AUC analyses appear consistent with findings from the DSM-IV Antisocial Personality Disorder field trial (Widiger et al. 1996) in which an index of affective and interpersonal features of psychopathy modeled after the PCL-R (which have been shown to reflect boldness along with meanness; Venables et al. 2014) failed to provide incremental prediction to clinical outcomes over and above the behaviorally-based DSM-III-R criteria set. Critically, however, psychopathic traits *did* provide incremental validity within the inmate subsample of the field trial (Widiger et al. 1996). Thus, although scores on TriPM Boldness did not provide information regarding group membership in the present study, boldness may moderate important clinical outcomes among those high in meanness and/or disinhibition (Miller and Lynam 2016) or contribute to phenotypic variance reflecting specific psychopathy subtypes or variants (Drislane et al. 2014b).

Current findings thus support the reliability and validity of the TriPM as a tool for assessing psychopathic tendencies. Importantly, the current results suggest that concerns about socially desirable responding and distorted self-perceptions affecting the validity of self-report based assessments of psychopathy may be overstated. Indeed, Edens et al. (2008) found that self-reported PPI scores actually outperformed clinician-rated PCL-R scores in the prediction of institutional misconduct. However, it should be noted that PPI administration in this work took place in a research context as opposed to a clinical evaluation context. Given the greater efficiency of self-report relative to interview-based instruments, questionnaire measures like the TriPM may provide a useful alternative tool for assessing psychopathy—perhaps especially in contexts where some degree of anonymity is assured.

Although current findings are promising, some limitations of the study should be considered when interpreting the results. First, the data were from two different samples tested in separate settings, which may have contributed to differences in findings between the community sample and forensic psychiatric samples. However, we think that this would be very unlikely to have occurred. Another limitation is that data for the PPI-R and RPQ were available only for a subset of each sample, further complicating comparisons of findings for the two samples. Additionally, the forensic sample was almost entirely male (93%); thus, it is unclear whether findings from the ROC curve analyses may partly reflect differences in gender composition of the two samples,^1^ and whether findings would generalize to female forensic patients.

However, the current study also has some notable strengths. Analyses utilized data from both community and forensic psychiatric inpatient samples, making it possible to compare outcomes for clinical versus non-clinical participants. Additionally, this study was the first to examine the discriminative ability of the TriPM compared to the PPI-R through ROC curve analyses, with findings supporting the effectiveness of the TriPM in discriminating between ‘healthy controls’ and forensic patients.

As such, the current study extends what we know about the properties and correlates of the TriPM operationalization of the triarchic model of psychopathy, and adds to a growing body of data pointing to the value of self-report based measures for indexing psychopathic tendencies in differing participant samples and settings.

## Footnotes

1 We conducted a ROC curve analysis for males only and found similar results.
